# Potential Implications of Rimonabant on Age-Related Oxidative Stress and Inflammation

**DOI:** 10.3390/antiox11010162

**Published:** 2022-01-14

**Authors:** Renáta Szabó, Zsuzsanna Szabó, Denise Börzsei, Alexandra Hoffmann, Zelma Nadin Lesi, Patrícia Pálszabó, Andrea Pálszabó, Szabolcs Dvorácskó, Rudolf Gesztelyi, Krisztina Kupai, Dániel Priksz, Béla Juhász, Anita Altmayer, Csaba Varga, Anikó Pósa

**Affiliations:** 1 Department of Physiology, Anatomy and Neuroscience, Faculty of Science and Informatics, University of Szeged, H-6726 Szeged, Hungary; szaborenata88@gmail.com (R.S.); szzsuzsi15@gmail.com (Z.S.); borzseidenise@gmail.com (D.B.); hoffmannalexandra1228@gmail.com (A.H.); lesi.zelma@gmail.com (Z.N.L.); palszabo.patricia@gmail.com (P.P.); palszabo.andrea@freemail.hu (A.P.); kupai@bio.u-szeged.hu (K.K.); altmayer.anita@gmail.com (A.A.); vacs@bio.u-szeged.hu (C.V.); 2 HR-Pharma Ltd., H-6726 Szeged, Hungary; 3 Laboratory of Chemical Biology, Institute of Biochemistry, Biological Research Centre, H-6726 Szeged, Hungary; dvoracsko.szabolcs@brc.hu; 4 Department of Pharmacology and Pharmacotherapy, University of Debrecen, H-4032 Debrecen, Hungary; gesztelyi.rudolf@pharm.unideb.hu (R.G.); priksz.daniel@pharm.unideb.hu (D.P.); juhasz.bela@med.unideb.hu (B.J.); 5 Albert Szent-Györgyi Clinical Center, Department of Medicine, Medical Faculty, University of Szeged, H-6720 Szeged, Hungary

**Keywords:** aging heart, inflammation, oxidative stress, endocannabinoid system, rimonabant

## Abstract

Over the last decades, growing interest has turned to preventive and therapeutic approaches for achieving successful aging. Oxidative stress and inflammation are fundamental features of cardiovascular diseases; therefore, potential targets of them can improve cardiac outcomes. Our study aimed to examine the involvement of the endocannabinoid system, especially the CB1 receptor blockade, on inflammatory and oxidant/antioxidant processes. Twenty-month-old female and male Wistar rats were divided into rimonabant-treated and aging control (untreated) groups. Rimonabant, a selective CB1 receptor antagonist, was administered at the dose of 1 mg/kg/day *intraperitoneally* for 2 weeks. Cardiac amounts of ROS, the antioxidant glutathione and superoxide dismutase (SOD), and the activity and concentration of the heme oxygenase (HO) enzyme were detected. Among inflammatory parameters, nuclear factor-kappa B (NF-κB), tumor necrosis factor-alpha (TNF-α), and myeloperoxidase (MPO) enzyme activity were measured. Two weeks of low dose rimonabant treatment significantly reduced the cardiac ROS via boosting of the antioxidant defense mechanisms as regards the HO system, and the SOD and glutathione content. Consistently, the age-related inflammatory response was alleviated. Rimonabant-treated animals showed significantly decreased NF-κB, TNF-α, and MPO levels. Our findings prove the beneficial involvement of CB1 receptor blocker rimonabant on inflammatory and oxidative damages to the aging heart.

## 1. Introduction

Aging is associated with a progressive decline in numerous physiological processes and is considered as an independent and cumulatively factor in the development and progression of cardiovascular diseases (CVDs). A large number of epidemiological evidence shows that a state of low-grade inflammation, termed inflammaging, makes the heart more vulnerable to cardiovascular injuries [1,2]. Even though the precise etiology and the diverse consequences of inflammaging are not fully elucidated, it is established that the accumulation of reactive oxygen species (ROS) leads to an imbalance between the antioxidant and oxidative systems, which can be associated with CVDs [3].

Over the last decades, there has been increasing attention to mitigating the risk of age-related abnormalities and achieving successful aging. There are various signaling mechanisms, which can be associated with systemic and tissue-specific antioxidant properties or even has a significant impact on inflammatory mechanisms. While nuclear factor-kappa B (NF-κB)-mediated pathways are responsible for the inflammatory response, the heme oxygenase (HO) enzyme system and glutathione serve as major regulators against oxidative damages [4]. Since its discovery, in the early 1990s, the endocannabinoid system (ECS) has increasingly emerged as a key signaling system for both physiological and pathological processes. Beside the central dominance, the cannabinoid 1, and 2 (CB1 and CB2) receptors are also expressed in peripheral tissues, including the myocardium and the vasculature [5]. It has been reported that the blockade of CB1 receptors contributes to the cardiac regulatory mechanisms as well as protects the heart against ischemia/reperfusion injury [6]. Among the antagonists of the CB1 receptor, rimonabant (SR141716) was the first antagonist to come into clinical use. Beside the cardiac mechanisms, several studies show that rimonabant plays an important role in different pathological conditions, such as atherosclerosis [7], obesity [8], non-alcoholic fatty liver disease [9], or nephropathy [10]. These previous studies have underpinned that oxidative/nitrosative stress and an increased inflammatory response serve as a common ground for the diseases. Whereas oxidative and nitrosative damages accelerate the course of diseases, blockade of CB1 receptors serves as a promising strategy to prevent adverse outcomes. Similar to diseases with low-grade inflammation, the inflammaging phenomenon is closely linked to oxidant/antioxidant disruption, thus ECS/CB1 blockade can be placed at the interface between aging and inflammatory processes. Although less information is available about the modifications of the peripheral ECS during aging, cross-talk among the nervous, endocrine, and endocannabinoid systems accompanying aging is revealed [11].

Therefore, this study aims to investigate the ability of ECS in the modulation of inflammation and oxidative stress in female and male aging hearts. We hypothesize that the CB1 receptor blocker rimonabant aims at inflammation and oxidative-sensitive targets, and these results can provide approaches to develop novel strategies for age-related adverse effects.

## 2. Materials and Methods

### 2.1. Animals and Experimental Protocol

In our study, 20-month-old (aging) female (*n* = 18) and male (*n* = 18) Wistar rats (Toxi-Coop, Hungary) were housed under constant temperature (20–22 °C) and humidity (40–50%) with a 12/12 h light/dark cycle. The animals were allowed ad libitum food and water. A part of both aging female and male rats were randomly assigned (maximum 9 animals per group) to receive rimonabant treatment. Rimonabant hydrochloride (Sigma-Aldrich Ltd., Hungary) was dissolved into 0.1% Tween 80 in distilled water, sonicated for 20 s, and the daily dose of rimonabant (1 mg/kg/daily once) was administered *intraperitoneally*. Animals who did not receive rimonabant injection were treated with the same volume of vehicle (0.2 mL). The body weight was measured at day 0 (initial body weight) and the end of the experiment (final body weight) as well. The results are presented in Table 7.

After a 2-week experimental period, rimonabant-treated and untreated rats were euthanized (100 mg/kg thiopental, B. Braun Medical SA, Barcelona, Spain) and sacrificed for biochemical measurements. Whole hearts were excised and then stored at −80 °C for further biochemical analysis. In our experiment, all efforts were made to minimize the number of animals as well as the animals suffering.

All procedures were in full compliance with the Directive of the European Parliament (2010/63EU) and were approved by the European Community guidelines on the care and use of laboratory animals (XX./2633/2020).

### 2.2. Determination of Cardiac HO Activity

Rat cardiac tissues were homogenized in ice-cold buffer containing 10.0 mM HEPES, 32.0 mM sucrose, 1.0 mM DTT, 0.10 mM EDTA, 10.0 μg/mL trypsin inhibitor, 10.0 μg/mL leupeptin, and 2.0 μg/mL aprotinin (pH 7.4). After centrifugation at 15,000× *g* for 20 min at 4 °C, the supernatant was discarded. The reaction mix contained 2.0 mM glucose-6-phosphate, 0.14 U/mL glucose-6-phosphate dehydrogenase, 15.0 μM hemin, 120.0 μg/mL rat liver cytosol as a source of biliverdin reductase, 2.0 mM MgCl_2_·6H_2_O, 100.0 mM KH_2_PO_4_, and 150.0 μL supernatant. The reaction was initiated with 100.0 μL reduced β-NADPH and then the reaction mix was incubated in the dark for 60 min at 37 °C. Bilirubin content was determined by optical density, which was measured at 465 and 530 nm, and the difference between the two densities was calculated. HO activity was defined as the amount of bilirubin (in nmol) produced per hour per milligram of protein.

### 2.3. Determination of Cardiac ROS, HO-1, NF-κB, and TNF-α Concentrations

Cardiac samples were homogenized in ice-cold phosphate buffer (pH 7.4) for 20 s. After centrifugation (20 min, 2500 rpm, 4 °C), supernatants were collected carefully and used for ELISA and protein measurements. According to the manufacturer’s datasheet (Gen Asia Biotech Co., Ltd., Shanghai, China), the amount of either 40 µL cardiac tissue supernatant or 50 µL standard solution was added to the wells that are pre-coated with ROS monoclonal antibody. Additionally, the supernatant-contained wells were completed with 10 µL of the second antibody labeled with biotin. Subsequently, 50 µL Streptavidin-HRP was also added to both the supernatant and the standard-contained wells, which consequently formed an immune complex with a biotin-labeled antibody. After the incubation procedure (60 min at 37 °C), the plate was washed 5 times; thus, the unbound enzymes have been removed. For the color development, 50 µL of substrate solution A and B were added to the wells and incubated for 10 min at 37 °C from light for color development. As the last step, 50 µL of stop solution was pipetted, which resulted in a change from blue to yellow with the effect of acid. The optical density was measured at 450 nm (Benchmark Microplate reader, Bio-Rad, Hercules, CA, USA). According to standard concentrations and the corresponding OD values, the linear regression equation of the standard curve was calculated. Cardiac ROS values were expressed in U/mg protein, while HO-1, NF-κB, and TNF-α values were in pg/mg protein [12].

### 2.4. Measurement of Cardiac Total (GSH+GSSG) Level

Cardiac tissues were homogenized in Homo buffer A (0.25 M sucrose, 20 mM Tris, and 1 mM dithiothreitol (DTT)) at first and then in Homo buffer B (0.1 M CaCl_2_, 0.25 M sucrose, 20 mM Tris, and 1 mM DTT). Discarded supernatant amounts were centrifuged for 30 min at 4 °C, and the remaining clear cytosolic fraction was used for the enzyme assay. In a 96-well plate, the following reagents were mixed: 20 μL 5,5-dithiobis-2-nitrobenzoic acid (DTNB), 140 μL nicotinamide adenine dinucleotide phosphate (NADPH), 10 μL glutathione reductase, and 40 μL sample. DTNB formation was determined at 405 nm, 10 min after the initiation of the reaction. GSH levels were expressed as nanomol per milligram protein.

### 2.5. Determination of Serum Superoxide Dismutase Activity

Serum samples were pipetted as duplicates into the plate (each sample had its blank modality). As described in the manual and our previous study [13], WST working solution, Dilution Buffer, Enzyme Working solution were added to the wells, then the plate was incubated at 37 °C for 20 min. Output was measured at 450 nm with a microplate reader. Data were analyzed as follows:

$$\mathrm{SOD}\;\mathrm{Activity}(\mathrm{inhibition}\;\mathrm{rate}\;\%)=\frac{(A_{\mathrm{blank}1}-A_{\mathrm{blank}3})-(A_{\mathrm{sample}}-A_{\mathrm{blank}2})\times 100}{\left(A_{\mathrm{blank}1}-A_{\mathrm{blank}3}\right)}$$

### 2.6. Determination of Cardiac MPO Activity

Heart tissues were homogenized in phosphate buffer (pH 6.0) containing 0.5% hexadecyltrimethylammonium bromide. Homogenized samples were placed first in liquid nitrogen than in a water bath (37 °C). These steps were repeated 3 times, then the samples were centrifuged (15,000× *g* for 15 min at 4 °C) and supernatants were collected. The measurement was performed on a 96-well plate. 280 µL of o-dianisidinediHCL along with 12 µL of sample or standard (diluted from peroxidase) were pipetted into the plate. Cardiac MPO activity was measured under 490 nm wavelength after 59 s of shaking and expressed as µU/mg protein.

### 2.7. Protein Determination

Using a commercial protein assay kit (Bio-Rad Labs), aliquots (20 μL) of the diluted samples were mixed with 980 μL of distilled water, with 200 μL Bradford reagent added to each sample. After mixing and following a 10 min incubation, the samples were assayed spectrophotometrically at 595 nm. The protein level was expressed as mg protein/mL.

### 2.8. Statistical Analysis

The normality of data was verified with the Shapiro-Wilk test. For comparison, two-way ANOVA with Tukey’s multiple comparison test was carried out. Statistical significance for the differences of means was assigned into one of five categories: *p* > 0.05 (not significant), *p* < 0.05 (*), *p* < 0.01 (**), *p* < 0.001 (***) or *p* < 0.0001 (****). Statistical analysis was carried out with the use of GraphPad Prism 8.4.3 software.

## 3. Results

### 3.1. Determination of Cardiac ROS Concentration

**Figure 1:**
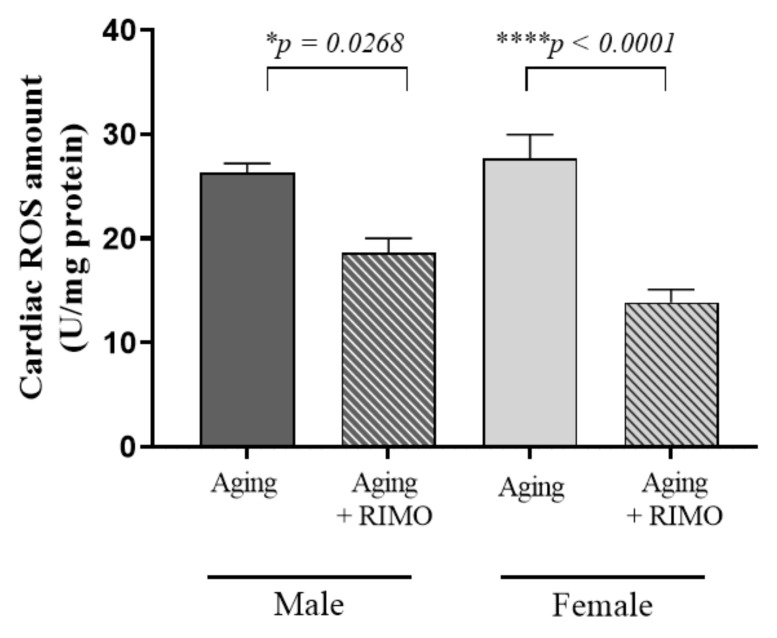
The effects of rimonabant treatment on cardiac ROS concentration in aged animals. (ROS; ex-pressed as unit/mg) Results are shown as means S.E.M. *n* = 5–6. * *p* < 0.05, **** *p* < 0.0001: Statistical significance between rimonabant-treated and untreated, sex-matched aging rats, RIMO = rimonabant, ROS = reactive oxygen element.

To investigate the potential involvement of rimonabant on aging-induced oxidative stress, the total ROS concentration was measured from cardiac tissue. We found that 2-week rimonabant administration significantly decreased the ROS concentration in both male and female rats, suggesting a beneficial effect of rimonabant on oxidative damage. Additionally, our results show that rimonabant treatment had a significant impact on the cardiac ROS content. Data are presented in Figure 1 and Table 1.

### 3.2. Measurement of Cardiac HO-1 Concentration and HO Activity

**Figure 2:**
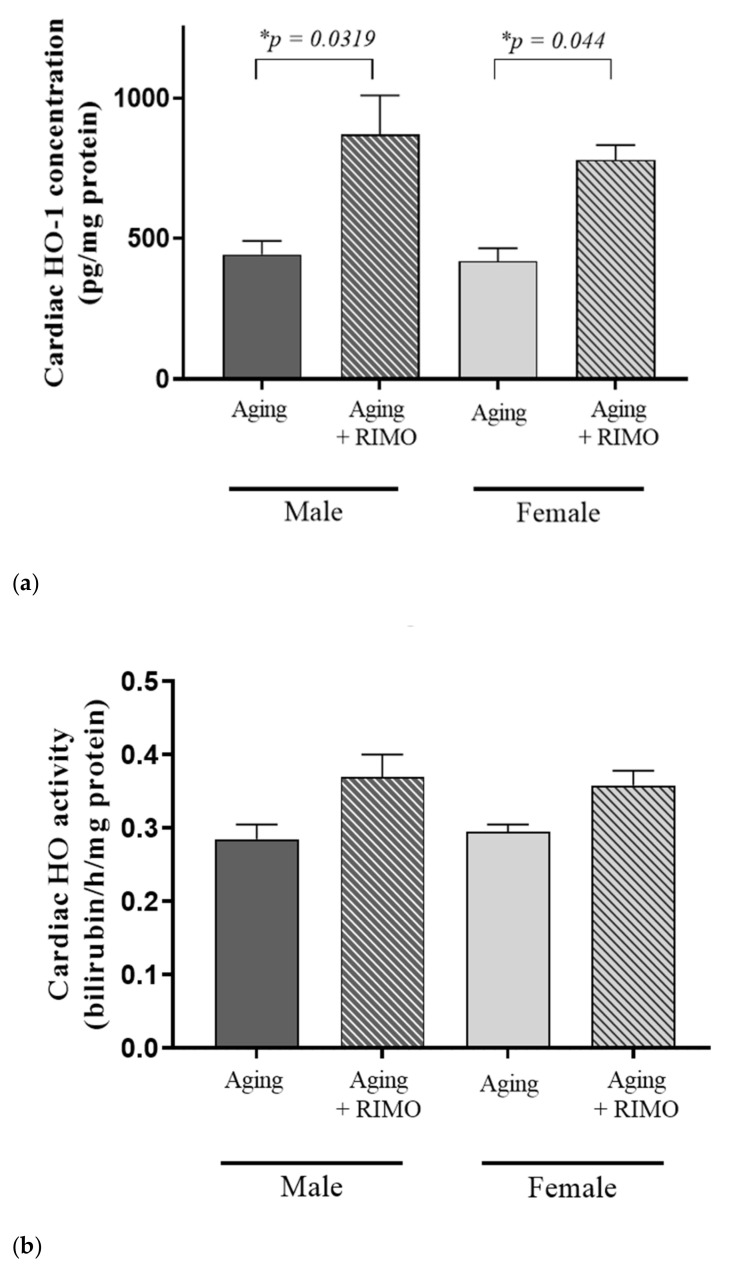
(**a**) The effects of rimonabant treatment on cardiac HO-1 concentration in aged animals (HO-1 expressed as pg/mg protein). Results are shown as means S.E.M. *n* = 4–7. * *p* < 0.05: Statistical significance between rimonabant-treated and untreated, sex-matched aging rats, RIMO = rimonabant, HO-1 = heme oxygenase-1. (**b**) The effects of rimonabant treatment on cardiac HO activity in aged animals. (HO; expressed as nmol bilirubin/h/mg protein). Results are shown as means ± S.E.M. *n* = 4–7. RIMO = rimonabant, HO = heme oxygenase.

At the end of the 2-week rimonabant treatment, the cardioprotective HO system was examined and evaluated whether rimonabant administration possesses ROS-scavenging properties. Figure 2 and Table 2 show that the CB1 blocker rimonabant resulted in a significant elevation of HO-1 concentration in both sexes and these findings were supported by the statistically positive independent effect of the treatment.

Although there is no significant difference in the level of HO activity between rimonabant-treated and untreated groups, our statistical data prove that the treatment itself has an effective role in the HO activity. HO activity data are presented in Figure 2a,b and Table 2a,b.

### 3.3. Determination of Cardiac Total (GSH+GSSG) Content

**Figure 3:**
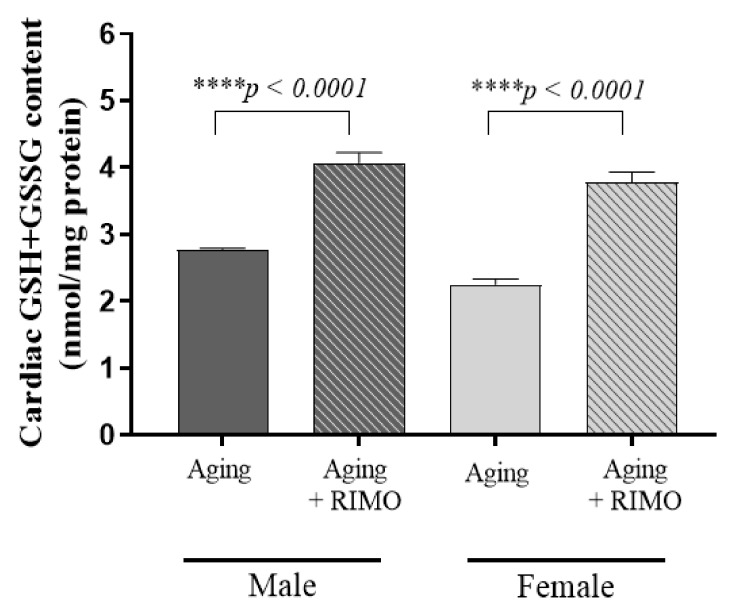
The effects of rimonabant treatment on cardiac GSH+GSSG content in aged animals. (GSH+GSSG; expressed as nmol/mg) Results are shown as means ± S.E.M. *n* = 5–8. **** *p* < 0.0001: Statistical significance between rimonabant-treated and untreated, sex-matched aging rats. RIMO = rimonabant, GSH+GSSG = total glutathione.

In relation to the antioxidant defense mechanisms, GSH+GSSG content was also measured. Our findings prove that 2 weeks of rimonabant treatment significantly increased the cardiac GSH+GSSG level compared to the untreated aging animals. Furthermore, there was sexual dimorphism in cardiac total glutathione levels between aging female and male rats. The results of the statistical analysis show that both aging and treatment are independent factors in the modulation of cardiac total glutathione content. Data are presented in Figure 3 and Table 3.

### 3.4. Determination of Serum SOD Activity

**Figure 4:**
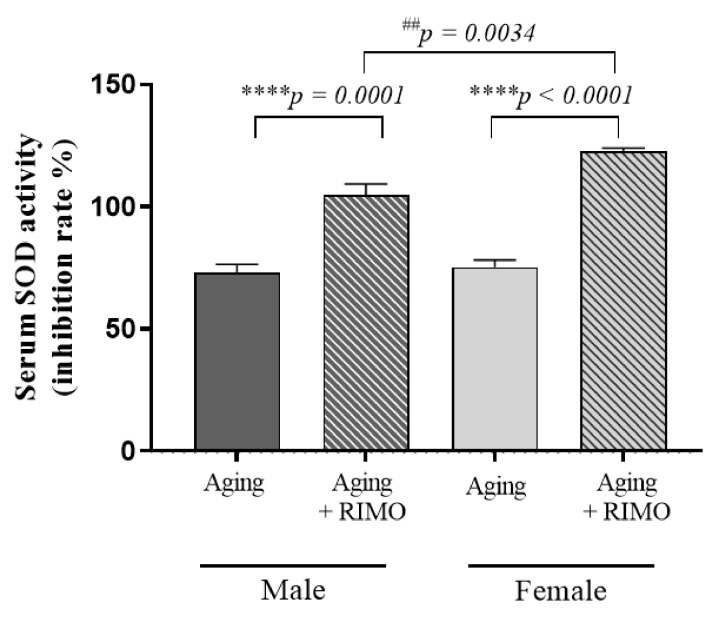
The effects of rimonabant treatment on serum SOD activity in aged animals (SOD expressed as inhibition rate %). Results are shown as means S.E.M. *n* = 6–8. **** *p* < 0.0001: Statistical significance between rimonabant-treated and untreated, sex-matched aging ^##^ *p* < 0.01: Statistical significance between the rimonabant-treated female and male aging rats. RIMO = rimonabant, SOD = superoxide dismutase.

To determine if a redox change systemically occurs as a result of rimonabant administration, serum SOD activity was measured. As shown in Figure 4, serum SOD activity was significantly elevated in rimonabant-treated female and male rats. Although the SOD values of untreated rats were similar between the untreated male and female counterparts, a statistically significant difference was detected in rimonabant-treated male rats compared to the female, treated rats. These results suggest that rimonabant treatment, in this scenario, serves a more potent antioxidant/SOD booster systemically.

Table 4 proves that both sex and treatment, either as independent factors or in interaction, possess a significant impact on systemic/serum SOD activity.

### 3.5. Measurement of Cardiac NF-κB and TNF-α Concentrations

**Figure 5:**
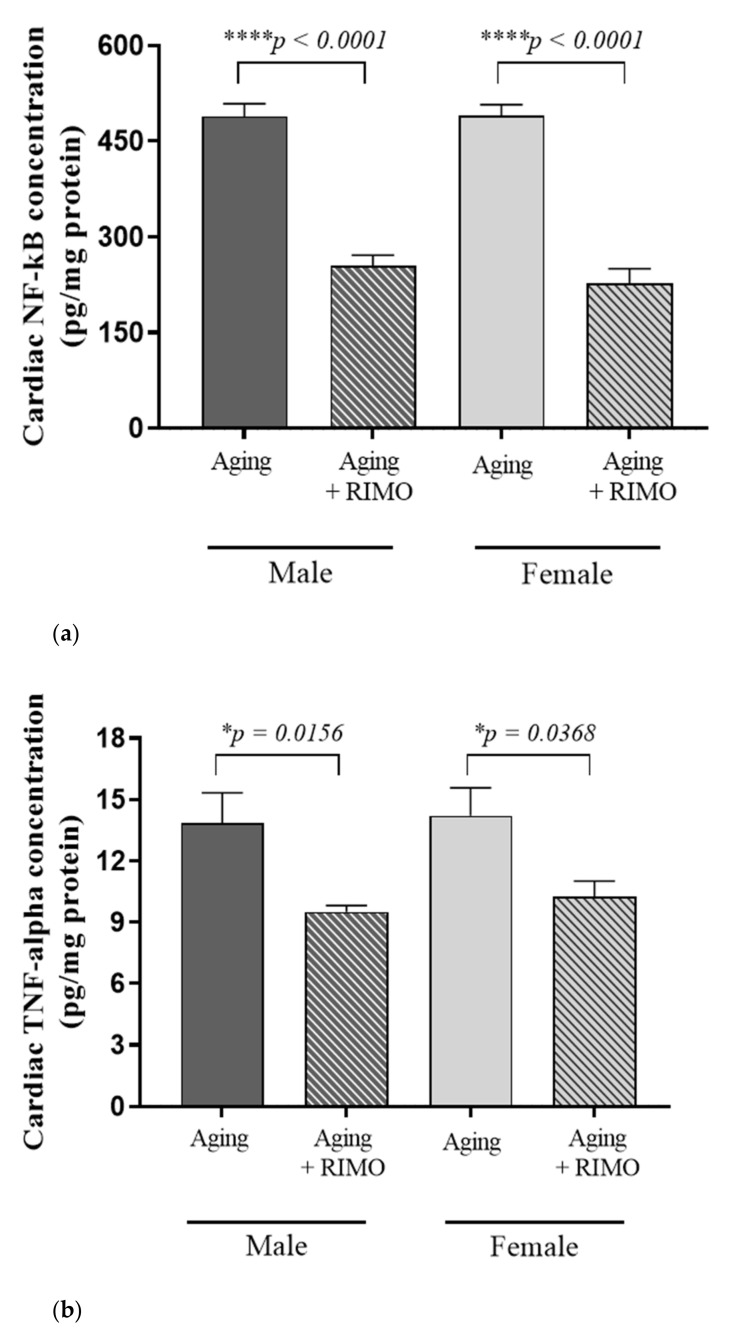
(**a**) The effects of rimonabant treatment on cardiac NF-κB concentration in aged animals. (NF-κB; expressed as pg/mg protein). Results are shown as means ± S.E.M. *n* = 6–8. **** *p* < 0.0001: Statistical significance between rimonabant-treated and untreated, sex-matched aging rats. RIMO = rimonabant, NF-κB = nuclear factor-kappa B. (**b**) The effects of rimonabant treatment on cardiac TNF-alpha concentration in aged animals. (TNF-α; expressed as pg/mg protein). Results are shown as means ± S.E.M. *n* = 5–9. * *p* < 0.05: Statistical significance between rimonabant-treated and untreated, sex-matched aging rats. RIMO = rimonabant, TNF-α = tumor necrosis factor-alpha.

As shown in Figure 5a,b and Table 5a,b, the cardiac concentrations of NF-κB and TNF-α were changed as a result of rimonabant treatment. We observed that 2-week rimonabant treatment significantly reduced the cardiac NF-κB concentration compared to untreated sex-matched aging animals. In accordance with the decrease in NF-κB, rimonabant caused a significant reduction in the pro-inflammatory TNF-α level in both sexes compared to the sex-matched aging animals. These findings suggest the potential anti-inflammatory properties of CB1 blocking. Our results present that treatment possesses a statistically significant impact on these inflammatory parameters.

### 3.6. Determination of Cardiac MPO Activity

**Figure 6:**
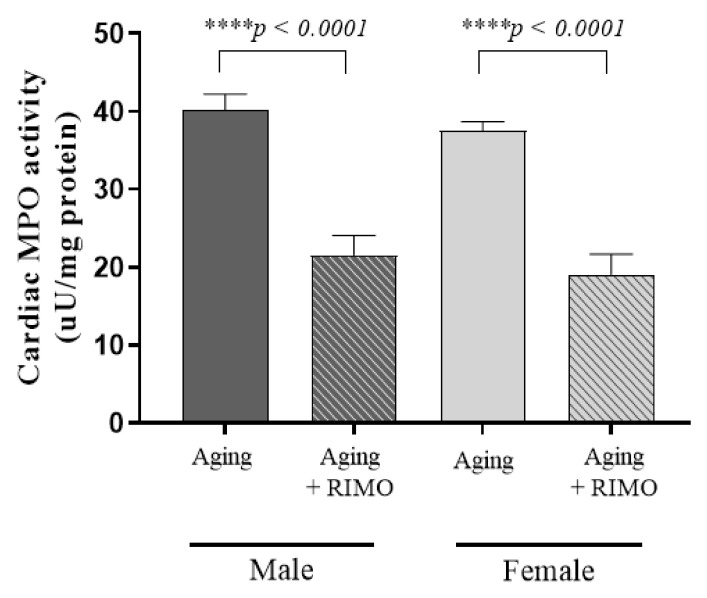
The effects of rimonabant treatment on cardiac MPO activity in aged animals. (MPO; expressed as µunit/mg protein). Results are shown as means ± S.E.M. *n* = 6–9. **** *p* < 0.0001: Statistical significance between rimonabant-treated and untreated, sex-matched aging rats. RIMO = rimonabant, MPO = myeloperoxidase enzyme.

Similar to the previous findings related to inflammation, rimonabant-treated rats disposed significantly of decreased MPO activities compared to the untreated sex-matched groups. Two-week rimonabant administration resulted in a ~54% decrease in male groups and a 51% decrease in female animals. Furthermore, the findings were supported by the statistically significant individual effect of treatment. Data are presented in Figure 6 and Table 6.

### 3.7. Assessment of Body Weight Changes

Table 7 shows that 2 weeks of CB1 receptor blocking rimonabant treatment did not cause significant bodyweight reduction in any experimental groups. In the case of aging female rats, rimonabant treatment resulted in a 5.9% body weight loss compared to the final body weight.

## 4. Discussion

One of the main challenges facing researches and medicine in the 21st century is to map and gain an accurate understanding of the underlying mechanisms of the unavoidable aging process. Interventions focusing on deceleration and mitigation of the age-related adverse effect can reveal significant preventive and therapeutic targets for CVDs.

Cardiovascular aging is associated with a wide range of functional, morphological, and molecular changes, which can culminate in severe cardiac outcomes [14,15,16,17]. In accordance with the literature, our previous studies showed that advanced age is an independent risk factor for CVDs in both males and females. Although the incidence of CVDs in females is usually lower than in males of reproductive age, sex differences appear at the onset of estrogen loss, which makes females more susceptible to various CVDs [18]. Considerable evidence shows that advanced age in both gender results in an imbalance between oxidative and antioxidant systems, increases ROS production, and eventually leads to oxidative stress and inflammation [1,19,20]. Our present results clearly show that the cardiac level of total ROS was similar in both males and females, which underpins the hypothesis that females lose their “advance based on sex” during aging. Elevated ROS level contributes to oxidant/antioxidant shifts; thus, the enzymatic and non-enzymatic defense systems disrupt. One of the most sensitive ROS-scavenging systems is the HO enzyme system, which possesses numerous beneficial properties. HO catalyzes the first and rate-limiting step in the degradation of heme to yield equimolar amounts of carbon monoxide (CO), ferrous iron, and biliverdin that subsequently converts to bilirubin by biliverdin reductase [21]. Both biliverdin/bilirubin and CO dispose of anti-inflammatory, anti-apoptotic, and antioxidant effects; thereby, the HO system is the main contributor to cell survival. Three isoforms of HO exist; HO-1 is an inducible form, whereas HO-2 and HO-3 are constitutively expressed. A growing body of evidence proves that aging-induced oxidative stress and inflammation significantly decrease the HO activity and the expression of HO-1 isoform compared with rats in reproductive age, whereas this diminished level can be compensated by natural or synthetic agents/interventions that possess antioxidant or anti-inflammatory properties [16,22,23]. The multifactorial regulation of the HO system provides important insight into the underlying mechanisms of diseases related to inflammation or oxidative stress, and into several therapies under investigation target factors regulating the HO system directly or indirectly. The ECS and its blockade can be considered as a relatively novel oxidant-sensitive regulatory pathway in the modulation of aging processes. The ECS, which consists of endogenous ligands/endocannabinoids, their receptors, and enzymes involved in the synthesis and degradation of endocannabinoids, is upregulated in CVDs, although there are paradoxical results in the mechanisms and outcomes of CB1 and CB2 agonists and antagonists. Rimonabant is the first-developed antagonist that has undergone extensive testing in the treatment of obesity because of its appetite suppressant role [24]. It selectively blocks CB1 receptors in the brain and peripherally; thus, it is not only an anti-obesity drug, but also limits vascular and myocardial inflammation, and exerts beneficial anti-inflammatory effects [25,26]. Although the clinical use of rimonabant has been withdrawn because of its psychiatric side effects, it is still an important drug to investigate the CB1 receptor-mediated pathways in different conditions.

In a previous study, Chang et al. demonstrated that CB1 receptor blockade via rimonabant administration ameliorated hepatic inflammation. They verified that the beneficial effects of rimonabant prevailed via increased mRNA level of nuclear factor erythroid 2-related factor (Nrf2) and increased gene expression of antioxidant protein levels, including HO-1 [27]. Nrf2 is a transcription factor, serves as one of the major regulators of the expression of proteins related to detoxification, reduction of oxidized proteins, and elimination of ROS [28]. Accordingly, the expression of HO-1 is largely under the control of the Nrf2 factor, which binds to the antioxidant response element (ARE) in the promoter region of the HO-1 antioxidant gene [29]. Satta et al. summarized that age-related reduction in Nrf2 results in oxidative stress or necrosis in the myocardium, which predisposes the heart to diseases; thus, activation of this factor can be a therapeutic strategy against inflammatory conditions [30]. Although Nrf2/ARE was not examined in our experiment, previous reports support the fact that the Nrf2/ARE complex is a significant target of the aging-induced ROS accumulation, which consequently results in a decrease in HO activity and expression. Focusing on this pathway, it can be concluded that rimonabant is a potent CB1 antagonist ligand, which successfully improves ROS-mediated oxidative damages. Two weeks of rimonabant administration significantly decreased the cardiac ROS accumulation in line with the elevation in HO activity and HO-1 concentration. Similar to the HO amelioration, total glutathione content was also enhanced in rimonabant-treated aged hearts. Beside HO, glutathione is a potent antioxidant that accounts for cardioprotective properties. The role of Nrf2 during oxidative conditions has been widely studied, compounds that can activate the Nrf2/ARE pathway, such as rimonabant in our study, plays a significant role in GSH-boosting in oxidative-related diseases. Whereas GSH depletion shifts the redox state towards prooxidant, ROS-scavenging interventions and mechanisms are able to compensate for the oxidative damage and provide a strong defense against oxidative/nitrosative injuries [31]. Similar to our findings, Jorgacevic et al. demonstrated that CB1 receptor blockade via rimonabant resulted in a significant glutathione release. These alterations were associated with the decrease of ROS, which can be the main source of inflammation- and oxidants-related pathological conditions, such as nonalcoholic fatty liver disease [9]. Beside glutathione enhancement, serum SOD activity was significantly increased as a result of 1 mg/kg rimonabant treatment. Elevated serum SOD level refers to the systemic antioxidant defense mechanism of rimonabant.

Additionally, the NF-κB-mediated pathway has been considered a prototypical pro-inflammatory signaling pathway, which is largely based on the activation of pro-inflammatory cytokines and ROS [32,33]. In a previous report, Lawrence summarized that the canonical NF-κB pathway is triggered by pro-inflammatory cytokines and it regulates cytokine production (e.g., TNF-α and IL-1); thereby, NF-κB serves an important target in inflammatory conditions [32]. Considerable studies suggest that the aging process corresponds with the inflammaging phenomenon that is underpinned by low-grade inflammation. Our findings have proved that cardiac senescence can be characterized by elevated levels of pro-inflammatory cytokines and myeloperoxidase (MPO) enzyme as well as diminished antioxidant capacity [18,34]. In this present work, 2-week-long rimonabant treatment contributed to the mitigation of TNF-α and MPO activity. Among several anti-inflammatory mechanisms, modulation of the ECS serves as a potential therapeutic strategy for inflammatory alterations. Confirming this hypothesis, Huang et al. demonstrated the inhibitory effects of rimonabant on TNF-α-induced NF-κB activation [35]. Based on the alterations related to Nrf2- and NF-κB-mediated pathways, it is highlighted that both pathways have an important role in rimonabant-associated defense properties. Growing evidence support that Nrf2 and NF-κB present yin and yang transcriptional forces in the maintenance of redox balance; although, the molecular and phenotypic outcomes are different. Suppression of NF-κB explains the anti-inflammatory effects of many activators of Nrf2- and its related pathways, whereas inactivation of this latter leads to loss of NF-κB suppression and results in inflammation [36]. Targeting of these pathways plays an important role in the reduction of age-related pathological alterations.

## 5. Conclusions

Beyond the well-known anti-obesity effects of rimonabant, its ability to promote anti-inflammatory and antioxidant alterations in different tissues has received great attention. Understanding the mechanisms that lead to inflammaging and oxidative stress, have important implication for the design to reduce age-related morbidity. Our results prove that the CB1 receptor blocker rimonabant has a significant role in the mediation of cardioprotective effects in aged female and male rats. Two weeks of rimonabant administration resulted in a significant decrease in the levels of inflammatory NF-κB, TNF-α, and MPO parameters as well as made the heart more protected from oxidative damages. The antioxidant HO and glutathione defense systems prove to be important ROS scavengers that were enhanced by rimonabant. It is demonstrated that the endocannabinoid system via CB1 receptor blocking alleviated the age-related adverse effects in both female and male animals; however, rimonabant-mediated advantage based on sex cannot be attributed to either aged group as regards the measured parameters. Although rimonabant has been withdrawn from the market, it is still an important compound as it is aimed at several targets to protect the heart against age-related damages.

## Figures and Tables

**Table 1:** Statistical table of the individual effects and interactions of rat sex and treatment on cardiac ROS concentration.

Source of Variation	*p* Value
Interaction	0.0862
Treatment	0.3285
Sex	<0.0001

**Table 2:** (**a**) Statistical table of the individual effects and interactions of rat sex and treatment on cardiac HO-1 concentration. (**b**) Statistical table of the individual effects and interactions of rat sex and treatment on cardiac HO activity.

(**a**)	
**Source of Variation**	***p* Value**
Interaction	0.7257
Sex	0.551
Treatment	0.0005
(**b**)	
**Source of Variation**	***p* Value**
Interaction	0.6506
Sex	0.9535
Treatment	0.0074

**Table 3:** Statistical table of the individual effects and interactions of rat sex and treatment on cardiac GSH+GSSG content.

Source of Variation	*p* Value
Interaction	0.3079
Sex	0.0036
Treatment	<0.0001

**Table 4:** Statistical table of the individual effects and interactions of rat sex and treatment on serum SOD activity.

Source of Variation	*p* Value
Interaction	0.0217
Sex	0.0047
Treatment	<0.0001

**Table 5:** (**a**) Statistical table of the individual effects and interactions of rat sex and treatment on cardiac NF-κB concentration. (**b**) Statistical table of the individual effects and interactions of rat sex and treatment on cardiac TNF-α concentration.

(**a**)	
**Source of Variation**	***p* Value**
Interaction	0.4548
Sex Treatment	0.4931 <0.0001
(**b**)	
**Source of Variation**	***p* Value**
Interaction	0.8328
Sex	0.5682
Treatment	0.0002

**Table 6:** Statistical table of the individual effects and interactions of rat sex and treatment on cardiac MPO activity.

Source of Variation	*p* Value
Interaction	0.9821
Sex	0.2522
Treatment	<0.0001

**Table 7:** Mean body weight values of male and female rimonabant-treated and untreated groups, and body weight changes measured between the initial and final days of the experimental period. RIMO = rimonabant. ↓: decrease of the parameter.

Body Weight Changes			
Groups	Mean Initial Body Weight (g)	Mean Final Body Weight (g)	Changes between the Final and Initial Body Weights (%)
Aging male	644.5	635.1	1.5 % ↓
Aging male + RIMO	641.3	622.4	3 % ↓
Aging female	317.3	321.0	1.2 % ↓
Aging female + RIMO	307.4	289.3	5.9 % ↓

## Data Availability

All data used to support the findings of this study are included within the article.
